# Cancer ecosystem assessment in West Africa: health systems gaps to prevent and control cancers in three countries: Ghana, Nigeria and Senegal

**DOI:** 10.11604/pamj.2020.35.90.18516

**Published:** 2020-03-25

**Authors:** Omobolaji Ayandipo, Issa Wone, Ernest Kenu, Luther-King Fasehun, Oluwayemisi Ayandipo, Fatou Gaye, Adedoyin Ojo, Yewande Ayoola, Jarim Omogi, Desta Lakew, Sylla Thiam

**Affiliations:** 1 Department of Surgery, College of Medicine University of Ibadan and University College Hospital, Ibadan, Nigeria; 2 Health Sciences Department, University Assane Seck of Ziguinchor, Ziguinchor, Senegal; 3 University of Ghana, School of Public Health, Department of Epidemiology and Disease Control, Accra, Ghana; 4 The Wellbeing Foundation Africa, Abuja, Nigeria; 5 African Field Epidemiology Network, Abuja, Nigeria; 6 Amref Health Africa, West Africa hub, 105 Sacre Coeur 3 Dakar, Senegal; 7 Department of Surgery, University College Hospital, Ibadan, Nigeria; 8 Amref International University P.O. Box. 27691- 00506; Nairobi, Kenya; 9 Amref Health Africa, Headquarters Langata Road PO Box 27691-0506 Nairobi, Kenya

**Keywords:** Cancer, prevention, control, needs assessment

## Abstract

**Introduction:**

Sub-Saharan Africa is experiencing a rapid epidemiological transition with the increasing incidence of Non-Communicable Diseases (NCD). Among these, cancer is one of the main causes of death in adults. This is a public health problem whose burden is unknown due to lack of statistical data. In addition, the already overburdened health systems are experiencing enormous constraints to address the problem with the double challenge of communicable and NCDs.

**Methods:**

The purpose of this evaluation was to assess the capacity and needs of health systems to prevent and control cancer. A cross-sectional study, using both quantitative and qualitative methods, was conducted between April 2017 and February 2018 in target countries, through in-depth interviews with key actors, direct observations and documents review. The WHO framework for health system strengthening with the 6 pillars was used for the gaps analysis.

**Results:**

Little priority is given to the fight against cancer because of low political commitment. Programs´ resources are very limited and there is a poor coordination of the actions. Human resources are insufficient, and most of them are concentrated in the capital city. This limits access to care with a late consultation of patients. Diagnosis and treatment services are expensive and generally paid by households. Finally, the unavailability of reliable data at national level hinders the decision-based evidence.

**Conclusion:**

There is an urgent need to create strong partnerships at national and regional levels to (i) Advocate for a strong political commitment; (ii) Strengthen the coordination of actions and create more synergy among stakeholders; (iii) Improve the quality and quantity of human resources; (iv) Extend universal health coverage to cancer and improve program funding; and (v) Set up cancer registries at national level.

## Introduction

In most countries in sub-Saharan Africa, the burden of non-communicable diseases (NCDs) is increasing rapidly whilst infectious diseases continue to pose major challenges [1], giving a double burden of disease to the sub-region. Cancers are among the major NCDs with high morbidity and mortality rates in Africa. According to the most recent Global Burden of Disease (GBD), NCDs constituted the greatest portion of deaths in 2017, at 73.4% - this represents a 22.7% increase from just ten years earlier in 2007 [2]. The global cancer statistics of 2018 had estimated that there will be 18.1 million new cancer cases and 9.6 million cancer deaths this year. According to the report, in both sexes, lung cancer is the most commonly diagnosed cancer (11.6% of the total cases) and the leading cause of cancer deaths (18.4%), followed by female breast cancer (11.6%), prostate cancer (7.1%) and colorectal cancer (6.1%) [3,4]. It is commonly suggested that the rise in incidence and deaths of non-communicable diseases is linked to poor socioeconomic status of individuals especially in low- and middle-income countries of Africa [5,6]. Late presentation, poor diagnosis, and lack of access to treatment are major contributory factors to poor prognosis in most developing countries [5,7]. In 2017, it was reported that pathology services were available in only 26% of low-income countries [5].

In a broad scope, developing countries, in particular African ones, are not ready to face the upcoming pandemic, with consideration to the rapidly increasing incidence of the NCDs, the weakness of the health system, and the very slow trends of the financial resource allocation [8]. Despite this threat, the burden of cancer is unknown in most of the African countries mainly because of a lack of data or under-reporting of cases. In addition, the national and subnational health systems in these countries face challenges such as inadequate diagnostic facilities, limited access to care, inadequate technical capacity and infrastructure, and all of these contribute to a weak response to the cancer burden. There is a dearth of reliable institutional data on cancers, so countries rely mostly on modelled data such as the GLOBOCAN (Global burden of cancer) report. Consequently, countries do not have the vital data needed to make informed decisions and policies [5]. This dearth of reliable and representative oncology data in Sub-Saharan Africa resulted in the World Health Organization (WHO) midwifing the GLOBOCAN project [9], which aims to provide on a regular basis, updated statistics on the incidence and prevalence of major types of cancer in 184 countries of the world, through combined sources including research and national statistics. To adequately plan prevention, diagnosis and management of cancers both at the community and health facility levels requires data. Amref Health Africa conducted a rapid needs assessment in Nigeria, Ghana and Senegal to better understand the current gaps in order to inform where to focus their attention towards the disease prevention and treatment. This paper assesses the needs and capacity of the three countries´ health systems to prevent and control cancers.

## Methods

**Study area:** the study was conducted in three West African countries; Nigeria, Ghana and Senegal with a combined population of about 235 million (190,886,311; 28,833,629 and 15,850,567 inhabitants respectively). The three countries are all members of the Economic Community of West African States (ECOWAS) a multicultural regional bloc with a population of approximately 367,255,391 inhabitants, spanning a variety of ethnic, linguistic and religious groups [10]. In Ghana and Senegal health systems organization is structured around three levels: national, regional and district. Ghana has 10 administrative regions with each having a regional hospital and 216 districts, while Senegal has 14 administrative regions (with 14 regional hospitals) and 76 health districts. On the other hand, Nigeria is a federal government system with 36 states and one Federal Capital Territory (Abuja), all of which combine the 774 local government areas (LGAs). In each country healthcare is delivered through various health facilities at primary, secondary and tertiary level. Institutions offering healthcare in Ghana includes 407 hospitals, 328 maternity homes, 1,858 clinics/ health centres and 4185 Community-based Health Planning and Services (CHPS) [11]. Senegal has hospitals 39 hospitals, 100 health centers, 1,458 health posts and 2,130 health huts [12]. And Nigeria counts 59 teaching hospitals and federal medical centers, 3,303 general hospitals and 20,278 primary health centers (PHCs) and health posts [13].

**Study design:** this was a cross sectional study conducted between May 2017 and February 2018 in target countries using both quantitative and qualitative approaches.

**Sample size and Selection of facilities:** relevant institutions were selected at the various levels (national, regional and district) of the health delivery system, according to their role in cancer control. In Ghana the two public comprehensive cancer centers at the two teaching hospitals (Korle-Bu Teaching Hospital KBTH, and Komfo Anokye Teaching Hospital KATH) and a private cancer center- Sweden Ghana Medical Cancer Centre (SGMC), with radiotherapy facilities were selected. Four (4) regional hospitals out of the 10 were selected and 4 district hospitals in each of the selected regions as well. At least one of the 4 district hospitals selected in the region was a private facility - overall, 23 health facilities were selected. In Senegal the assessment was conducted in all hospitals having a cancer unit or activity. These include 4 referral/teaching hospitals (Le Dantec, Grand Yoff, Fann and Principal) in Dakar and one regional hospital (Thies). In Nigeria, 6 geo-political regions, spanning two states per region (11 states), except Borno State (due to the insurgency) were assessed. The most established specialist referral hospital rendering specialist oncology care, in each geopolitical zone, was purposely selected. In each country key stakeholders in the NCDs´ Control Program in the respective Ministry of Health, and stakeholders in civil society organizations (CSOs) involved in cancer prevention and control were also interviewed.

**Data Collection:** a standardized questionnaire designed using the WHO framework for health system *(Capacity assessment questionnaire for the prevention and control of cancers)* [14], was developed and used to assess the key thematic areas. The questionnaire was divided into different sections for easy response filling, and in a chronological order. The questionnaire was developed in a user-friendly manner such that it could be self-administered and interviewer-administered. The assessment protocol was language-translated to French before adoption in Senegal. The Heads of the selected facilities or units and cancer specialists (medical oncologist, radiation oncologist, oncology nurses, pharmacist, nutritionist, psychologist, social workers) were interviewed. The various experts helped in completing the relevant sections of the tools, based on the thematic areas. Research assistants were trained in the use, interpretation and recording of the field tool. Activities included collection, transcription and summarizing qualitative data gathered through Key Informant´s Interview (KII), health facility assessment, record reviews, and observations. Completed questionnaires and check-lists were reviewed for quality, completeness, consistency and coherence, in order to ensure good quality data was generated, entered and analyzed. Desk review of literatures and documents was done in all countries.

**Data analysis** The quantitative and transcribed qualitative data were subsequently analyzed. Data analysis, triangulation, encoding transcription and interpretation for all countries were conducted. In Nigeria, each question was coded, entered and analysed using the Statistical Package for Social Sciences (SPSS) version 20.0 software while Atlas to version 7 was used for the qualitative analysis. The data in Ghana was entered into EPI info version 7 and was analysed using Stata version 13.0. Whereas in Senegal, qualitative data analysis was undertaken manually using responses reduction approach and quantitative data was managed using the Excel.

**Ethical considerations:** approval was obtained from the respective countries. Informed consent was obtained from each respondent after explaining to them the purpose of the study and their liberty to choose to participate in the survey, or not. Confidentiality was maintained as anonymity of respondents was ensured.

## Results

**Leadership, management and governance:** cancer, just like any other non-communicable disease, is yet to get a viable policy action from the government of the countries assessed. The Nigeria´s Federal Ministry of Health (FMoH) executes cancer policy through the National Cancer Control Programme (NCCP), which is more like a cancer department and does not have sufficient funding and capability to comprehensively control cancer in Nigeria. There is an operational unit for non-communicable disease within the Ministry of Health at the national and state levels in Nigeria. All the 11 State ministries of health visited had an NCD unit with just 1 officer in charge of the unit. Most of the units are not active and do not provide the overall leadership for prevention and control of cancers in the States. In August 2017, the Federal Ministry of Health convened stakeholders and together they agreed on the strategy dubbed National Cancer Control Plan (2018-2022) which has recently been launched. The Ministry of Health´s level of commitment previously to cancer control in Ghana was very low, but with continuous advocacy and education, the government commitment has improved. In September 2008, a National Cancer Steering Committee was established to advise the Government on all aspects related to cancer; oversee the establishment and operation of a National Cancer Control Programme; and develop policy guidelines on the implementation of the National Cancer Control Programme. The Ministry of Health, under the National Cancer Control Steering Committee developed a National Strategy for Cancer Control in Ghana, 2014- 2018. The program implementation is however, coordinated by the National Cancer Control Focal Point under the Non-Communicable Diseases Control Program. The office works with the various regional health directorates and the teaching hospitals to implement the cancer control strategy. There is a clear strategic direction outlined in the document for cancer control and prevention for a 5-year period, but the program is thus under resourced in both human and financial resources to implement their planned activities. The Ministry of Health has just developed the National Guideline for Cancer management dated February 2017. The Guideline focuses on the major cancers in Ghana namely: Breast, Cervical, Prostate, Non-Hodgkin Lymphoma and Childhood Cancers. This document is yet to be disseminated to all health facilities in the country. It describes the screening and early detection technique, the diagnostic techniques including the staging and the various treatment modalities (chemotherapy, radiotherapy and surgery) for the various cancers. Senegal has already put in place an NCDs Programme with only five (5) employees, though with none of the staff assigned to exclusively work on the prevention and control of cancer. However, the country has developed two main documents that are geared towards management of NCDs and cancer. These are *The Integrated Plan for the Fight against Non-Communicable Diseases (2017-2019) and The Strategic Plan for the Fight against Cancer (2015-2019)*. Unfortunately, these documents are not fully implemented yet, due to lack of funds. Besides, there is no national cancer steering committee to guide, coordinate and monitor the various interventions in Senegal. There are no evidence-based national guidelines /protocols/standards for the management of cancer through a primary care approach.

**Health care financing:** the management of cancers in the countries assessed is faced with major issues regarding funding. Out-of-pocket expenditure remains quite high while there is limited insurance coverage for treatment of cancer. Inadequate funding is a major challenge in healthcare service delivery in Nigeria. Apart from an NGO (Non-government organization) called Freedom for Life Initiative that partnered with one of the facilities and treated some children with cancer, all the centers said they are not getting any funds and, most specifically, the National Health Insurance Scheme (NHIS) was not adequately covering cancer management. There have also been cases where doctors donate their services in collaboration with hospital-based welfare or social funds (such as the Alaanu Fund in University College Hospital, Ibadan), to help very needy patients. In Ghana, the recent major investment in the area of cancer control by the government was a loan of US$13.6 million secured to upgrade the KBTH and KATH cancer centers. The upgrade included the procurement and installation of Linear Accelerators and other modern cancer treatment equipment. The commitment, however, has been mainly in the area of cancer treatment at the neglect of cancer prevention (screening and early detection). This has resulted in patients presenting to the facilities at the advanced stage, and hence leading to poor treatment outcomes. Palliative care has also been neglected. Apart from the capital investment in cancer treatment centers, there has been no resource allocation from the government in cancer prevention and control, including awareness, early detection and screening, palliative care and cancer registration. The Non-Communicable Diseases Control Program (NCDCP), which hosts the office of the cancer control focal point for the Ministry of Health, does not have a budget, and hence does not receive any funds either from government or donors for cancer prevention activities. The program is thus under resourced in both human and financial resources to implement their planned activities. There is a national Health Insurance Scheme that caters for some of the cancer diagnosis and treatment of patients who are insured. There is a National Health Insurance Schemes covering all regions in Senegal and operating in public facilities, but it has not included cancer screening and treatment in the services package. No stable resources allocation is dedicated to cancer at the country scale. The funds are mainly provided by NGOs and foundations, on a very sporadic basis.

**Health workers in cancer care:** a total of 372 respondents were assessed in Nigeria, while there were 122 respondents in Ghana, and a total of 42 in Senegal, as seen in Table 1. Across the three countries majority (2/3) of the health workers working in oncology units did not have formal training in oncology but they received guidance in the field of oncology. All oncologists´ specialists who participated in the study in Nigeria had a primary medical degree, about a third were in the post graduate training program, while 64.4% of them had further training that led to the acquisition of post-graduate fellowship. In Ghana 39 respondents were medical doctors with varied expertise in cancer care and different years of experience. There were those with certified medical and clinical oncologist with expertise or knowledge in radiotherapy and nuclear medicine. The rest especially in the regional and district primary and secondary level facilities only acted as first point of call in making cancer diagnosis. The trained oncologists were limited to the two teaching hospitals and the private cancer medical centre. The rest of the medical officers provide some cancer services in their facilities, but they are not trained oncologist. Of the 8.2% of health workers who had formal training in oncology, majority were trained in Ghana at the School of Nuclear and Allied Health, University of Ghana with just a few who trained in South Africa. In Senegal the human resources deficit not only affects the oncologists but other cadres too. There was a dozen of oncologists all located in the capital city. The specialists were trained at the National Cancer Institute in Senegal or by other institutions in France. Further-more more than half of the respondents noted that there was no ongoing training in oncology in their department/hospital/clinic with all emphasizing on the advantages of an additional training activity. Almost one third (31.3%) of the respondents in Nigeria saw lack of equipment/facility as the biggest challenge they face in their work with oncology patients followed by non-diagnosis/treatment acceptance (25.5%). In Ghana, major challenges faced included: late presentation to the hospital, non-availability of cancer medication, lack of family support/neglect, patients not able to afford medications, inability for the facility to diagnose the condition, patient refusing referrals, language barrier, loss to follow up, improper documentation and filing of patient folders, non-compliance, non-acceptance of diagnosis, work load etc. According to the respondents in Senegal, the major constraint to their service to patients included: accessibility and cost of treatment, late presentation of the cancer patients, inadequate infrastructure, acute shortage of health workers, lack of specialized and adapted psycho-social care, among others. Most respondents (68.2%) in Nigeria were moderately satisfied with their work in oncology, and about 77.10% had moderate knowledge of oncology. Similar findings were seen in Senegal with 78% and 71.4% satisfied with their work and had adequate knowledge in oncology respectively. In Ghana however, majority were in different about their feeling going to work in an oncology work environment although they thought knowledge was adequate.

**Table 1:** Distribution of health workers who participated in the assessment per country

	Nigeria	Ghana	Senegal
**Current profession**	n= 372	n=120	n=42
Nurse	66 (17.7)	65 (54.2)	21(50)
Health officer	60 (16.1)	1 (0.8)	0
General practitioner (MD)	78 (21)	9 (7.5)	4 (9.6)
MD Oncologist	133 (35.8)	7 (5.8)	13 (31)
MD Internist	29 (7.8)	2 (1.7)	1 (2)
Other	6 (1.6)	36 (30)	3 (7.1)
**Formal Oncology Qualification**	N=372	N=106	N=44
Yes	94 (25.3)	10 (8.2)	12 (27.3)
No	278 (74.7)	96 (78.7)	32 (72.7)

**Service delivery:** most healthcare professionals said that a majority of the patients presented themselves for treatment at advanced stages and most of the patients started cancer treatment late. On treatment interruptions, most of the oncologists recognized the breakdown of radiotherapy machines, among other reasons, (Table 2) as the main reason why treatment is interrupted. Other reasons were because patients could no longer afford treatment, the unavailability of chemotherapy and side effects of the drugs. In Nigeria and Ghana, a referral mechanism existed for referring cancer patients and their family. In the private cancer centre in Ghana, they followed up on their patients using phone calls, even though they did not have a direct system to provide community support. Senegal lacks guidelines on referral of cancer patients in the respective health system structure. Furthermore, the country lacks an organized system for monitoring, prevention, screening, diagnosis and treatment of cancer.

**Table 2:** Reasons for treatment interruption by patients according to the care providers

Cause	Nigeria estimated rate (%)	Ghana Estimated rate (%)	Senegal estimated rate (%)
Radiotherapy machine failure	43	6.2	42
Inability to continue treatment for financial reasons	27.9	21.3	42
Lost to follow-up for unknown reasons	17.9	8.4	21
Side effects	2.4	32.4	17
Out-of-stock medication	9.8	18.7	12
Preferred traditional medicine	7.3	37.9	2

**Medical products and technologies:** there is a limited chain of supply for cancer drugs. The study also found that only the tertiary hospitals had all the drugs needed, unlike the other levels of health care. In Nigeria in all sites visited, there is no chain supply for cancer drugs at the State level. Most of the tertiary hospitals assessed had a dedicated pharmacy for oncology where all needed drugs were available. The drugs that require cold chain were found to be kept with the suppliers who made them available as at when they were needed. Additionally, treatment is not free and drugs are purchased on a cash basis. In Ghana, outpatient services for providing medications for cancer diagnosis and treatment are limited only to the teaching hospitals and private cancer centre. The teaching hospitals have the opportunity of harnessing the capabilities embedded in other departments and thus even if the service is not in the cancer centre, it was available in other departments within the teaching hospital. The private cancer centre provides purely out-patient services The oncology drugs were readily available in the private medical cancer centre and the teaching hospitals providing cancer services. Antibiotics were readily available in all the health facilities. Nearly full range of radiotherapy services were available in the teaching hospitals and the private cancer centre. All facilities had available beds for inpatient care services and were in the position to admit cancer patients. Histopathology services were available only in the teaching hospitals. In Senegal despite the cost, the government of Senegal avails the drugs on the National List of Essential Drugs and Products (NLEDP). The National Medical Store has introduced first-line anticancer drugs since 2016 though the drugs are not always available in hospitals. Furthermore, patients missed their radiotherapy sessions since the only radiotherapy machine in the country had broken down for almost a year. Fortunately, significant progresses are ongoing, with the recent acquisition of two new radiotherapy machines, Drug storage seems to be a challenge given that some of them need to be stored in cool places. Though this is done by the supplies and are only availed on request, it may turn to be a challenge since the patients have to wait for the commodities to be ordered, hence increasing the waiting time of the patients.

**Health management information system (HMIS) and cancer registries** The National HMIS in Nigeria captures data from PHCs and secondary health facilities only. There is also an attempt to capture data from the tertiary centers, though this in most cases is incomplete. The study also realized that there are no cancer-related reports being collected in the NHMIS which feeds into District Health Information System (DHIS2) simply indicating that cancer-related data are not captured at the primary health care level. It was however encouraging that most centers had a cancer registry though not fully computerized. In Ghana, the national HMIS (DHIS2) and the two institutional Cancer registries in Ghana (Korle-Bu Teaching Hospital Cancer Registry and the Komfo Anokye Teaching Hospital Cancer Registry) were used. However, similar problems of a lack of national database and poor coordination in cancer data management were noted. The cancer registry in Senegal is considered as the reference tool for recording cases, and the source for cancer information. The information system of the cancer registry was developed by NCI with the Windev software. The Dakar project launched in 2009 was to collect information on cancer. The project worked closely with 10 hospitals. It is, however, sad that the registry collected incidence of different cancer types in 2010, and stopped functioning in the year 2014, due to lack of finances.

## Discussion

**Leadership/governance:** the Ministry of Health in these three countries are yet to fully implement a concrete policy action plan, thus making the fight against cancer haphazard and that which lacks effective coordination. The nonexistence of an institute to drive comprehensive cancer prevention, screening, palliative care and registration hinders effective oversight, accountability, coalition building with other partners, and appropriate regulations and incentives. The lack of an operational multi-sectoral entity in dealing with the NCDs is also a barrier to dealing with the disease. The major components of the action plans which include improving the HMIS to capture NCD data and coordinating partnership in prevention and control of NCD are yet to be implemented.

**Health care financing:** the Abuja Declaration [15], that at least 15% of Nigeria´s Gross domestic product (GDP) be allocated to health is yet to be implemented. In any case, there have been mixed signals on the amount to be allocated over the years with 2007 recording the highest allocation of 4.47%. The prevention and control of cancer is expensive and requires every partner and stakeholder´s effort when it comes to its financing. The financial catastrophe and impoverishment associated with cancer treatment and care is well documented in this study. This finding is consistent to a prior study which reported that though Nigeria is country that is rich with natural resources; federal hospitals have insufficient health care budgets to improve their radiotherapy delivery capacities [16]. Occasional capital investment in treatment centers, with little or no resource allocation for cancer prevention, control, early detection, screening, palliative care and awareness is the norm in Ghana. The Non-Communicable Diseases Control Program (NCDCP) which hosts the office of the cancer control focal point for the Ministry of Health, does not have a budget and hence does not receive any funds either from the government or donors, for cancer prevention activities. There is a national Health Insurance Scheme that caters for some of the cancer diagnosis and treatment of patients who are insured. The program is thus under-resourced in both human and financial resources to implement their planned activities. Cancer prevention and control financing remains a big impediment to the Government of Senegal in dealing with the burden of cancer. A few non-governmental organizations are on record to be doing screening, but all this is not well coordinated. Out-of-pocket expenditure remains quite high, while there is no existence of an insurance scheme for the treatment of cancer. Our findings are consistent to a study in Ghana where about five of the women took loans to finance their healthcare. The women borrowed between GHC100.00 (25 USD) and GHC6, 000.00 (1500 USD). They took loans from both formal (banks and microfinance) and informal source (friends and relatives). For those who took loans from microfinance institutions, they had to pay about 40 per cent interest on the loans they took. Some women lost their jobs and/or had a reduction in income; yet, had expensive medical bills to pay. Some respondents sold their assets and took loans with huge servicing and became indebted to the bank [17].

**Health workforce:** in Senegal, health workers are concentrated in specific urban centers particularly Dakar. For instance, Dakar region has 0.2 physicians per 1000 inhabitants while Fatick, Kaolack, Kolda, and Matam regions have fewer than 0.04 physicians per 1000 inhabitants. According to experts, Senegal´s major human resources for health (HRH) challenges include suboptimal coordination between public and private HRH actors and unemployment of health workers [18]. According to WHO, a well-performing health workforce is one which works in ways that are responsive, fair and efficient to achieve the best outcomes possible, given available resources and circumstances. According to our study, there is need for more health workers while those available need to be well distributed. Indeed, Ghana suffers from a chronic shortage of health workers as well as inequalities in both the distribution and skills mix of workers, and this severely restricts access to service and hampers achievement of national health objectives. The country has just over 11 doctors, nurses and midwives per 10,000 population, less than half the number (23 per 10,000) deemed necessary by the WHO for achievement health Goals. Rural areas, in comparison with urban areas, are particularly poorly served as regards access to health care; in 2009, for example, there was one doctor for every 5,103 people in Greater Accra, compared with one doctor for every 50,751 people in Northern Region [19]. In addition, there is a need to train more oncologists in order to reduce the acute shortage of health workforce in the country. Cancer care and treatment requires support from other cadres. With only a few of the workers having undergone oncology training, much needs to be done to increase this number, given the ratio of doctor to patient. Motivation is key in keeping the workforce happy and making their work productive. Most of the oncologists rated their satisfaction as moderate. This may be due to overworking, lack of equipment, poor working environment, among other reasons that may be beyond this study. The level of knowledge among the oncologists was also rated as moderate. This could be as a result of inadequate periodic training and lack of opportunities to further their knowledge.

**Medical products, technologies:** there is a limited chain of supply for cancer drugs, and this is worrisome. The study also found that only the tertiary hospitals had all the drugs needed, unlike the other levels of health care. Drug storage seemed to be a challenge given that some of them need to be stored in cool places. Though this is done by the supplies and are only availed on request, it may turn out to be a challenge, since the patients have to wait for the commodities to be ordered, hence increasing the waiting time of the patients. However, in a document by Brunner and others, Senegal is said to be having a well-structured and well-regulated systems for importing, manufacturing, storing, and distributing pharmaceuticals and medical commodities. The lead agency for regulation is the *Direction de la Pharmacie et Medicament* (DPM) which governs both the public and private sector supply chains, issues authorizations to import or manufacture pharmaceuticals products, and oversee drug assurance [18]. One of the biggest challenges to cancer diagnosis and treatment is the lack of facilities and equipment for treatment and diagnosis of cancer. Though availability and affordability scored average in the quantitative analysis, a lot needs to be done if the expression of the health workers in the qualitative analysis is anything to go by. Majority of the centers lacked radiotherapy machines and nuclear medicine services. This shows a picture of a poor functioning health system, thus comprising on the quality, safety and efficacy of cancer treatment. This is similar to a study in Nigeria in which it was found that management of patients with breast cancer is a major challenge to physicians with factors like lack of advanced technology (diagnosis and monitoring), poor access to cancer medication were cited [20]. In another study carried in Central Nigeria, the researchers noted that their institution did not have facilities for chemo-radiation and did not have a trained medical or gynecologic-oncologist in cervical cancer treatment. Due to this limitation, cervical cancer patients received either symptomatic care (including correction of anemia, treatment of infections, and pain control) or referral to Ahmadu Bello University Teaching Hospital (ABUTH), Zaria for chemo-radiation [21].

**Service delivery:** our findings show a lack of effective service delivery to the thousands of cancer patients across these three countries. Lack of proper and legally binding partnerships with other NGOs both at the grassroots and at the national levels hinder quality personal and non-personal cancer prevention and treatment interventions to the populace. Lack of well-trained personnel and inadequate infrastructure in the public hospitals is a barrier to quality service delivery. This has led to poor adherence to medication due to high cost of treatment in the private health centers leading to high morbidity and mortality. This is similar to a report in Ghana where significant disparities of service between north and south and between rich and poor, factors such as cultural and religious beliefs, poor physical infrastructure and limited resources were cited as barriers to quality health for all people living in Ghana [19]. Service delivery should be of high quality, and be offered when and where it is needed, with minimal wastage of resources. This is not presently the case, according to our findings, as a lack of radiotherapy machines and consultation rooms makes patients travel long distances to seek treatment, while others are forced to wait for longer hours to see a health worker or to be attended to. According to Diop *et al*. the high concentration of care offered in urban areas in Senegal and the high cost of provided services are to blame for the lack of access to care [22].

**Information and research:** the fact that cancer registries are not fully functional in the three countries and they capture only data from some secondary and tertiary health facilities only is a huge gap. As a result, much cancer cases go unreported. The cancer information system in these three countries is largely paper-based. This leads to bulk in documentation, and time wasting in collection, collation, analysis and reporting. This also leads to numerous errors that can be controlled by computerizing the system. Our results are similar to a study by Zelle *et al*. in 2014 where it was reported that the national cancer registry in Ghana is not fully functional and local data on breast cancer stage distribution were derived from Korle Bu Teaching Hospital, Accra and Komfo Anokye Teaching Hospital, Kumasi [23]. Since this is based on presenting patients rather than on all patients, this may not reflect reality and may have biased our estimates. Secondly, information on the epidemiology of breast cancer was not locally derived but based on the Globocan data and observations in other countries. A computerized system will lead to timely collection, analysis, dissemination and use of reliable and appropriate information on cancer. It will also lead to better coordination of cancer related information from different states.

## Conclusion

The epidemiology and types of cancer seen during this assessment is similar to those in other parts of the world, with increasing disease and death burden [24,25]. The coordination of cancer control is yet to be fully implemented in these three countries since their respective Ministry of Health developed a policy action plan, thus making the fight against cancer haphazard and that which lacks organization. The lack of an operational multi-sectoral entity in dealing with the NCDs is also a barrier to dealing with the cancer in these countries. There is an acute shortage of health personnel for cancer care, with a very high patient to cancer specialist ratio, and little or no specialists in the rural communities [26]. Majority of the health personnel available have no formal oncology training. Brain drain and increased sickness absenteeism [27], according to the literature, have deleterious effects resulting from the shortage of healthcare workers, and could possibly lower the quality of care offered to patients on cancer care. There is a lack of well-coordinated supply chain for drugs in addition to inadequate infrastructure across the states. A lot of cancer data is yet to be captured across the countries since NHMIS only captures information from PHCs and secondary health facilities on communicable diseases. The information system is largely paper-based making the activity cumbersome and prone to errors. There is a lack of effective service delivery to the thousands of cancer patients across the countries. Poor coordination with other partners, lack of well-trained personnel and inadequate infrastructure in the public hospitals are barriers to quality service delivery. In summary, little awareness, late presentation with limited access to cancer screening, diagnosis and treatment is a hallmark of cancer in these countries assessed.

**Recommendations:** considering all of the aforementioned, the following recommendations should be considered: high level advocacy to increase political will and commitment towards cancer prevention and control; strong partnerships and well-coordinated activities are key to combating cancers whose incidence and prevalence are on the rise. This also calls for community participation including the local leaders as has been documented as a vital strategy; creating awareness on the usefulness of early screening, and to bridge the gap in cancer care among patients; development and effective implementation of a national protocol for cancer prevention and screening is also equally advised. National protocols can be used as a monitor for choosing the method of screening for each individual, and these should be patient-centered, facilitating decision-making of cancer-screening services. Considerable effort should be put to ensure that national guidelines offer strategies based on the risk susceptibility of individuals [28]. This will aid delivery of holistic care for patients, bearing in mind the peculiarities associated with the different severities in the different stages of cancer; establishment of a sustainable and supportive financial platform for cancer treatment. Adequate finance is needed to promote value-based healthcare [29], subsidization of drug costs, and also for limiting the occurrences of stock-outs in pharmacies of the tertiary facilities. The role of innovative healthcare financing in global public health delivery is as apt for NCDs and cancer as it is for infectious and neglected tropical diseases (NTDs). In this regard countries should take the opportunity of universal healthcare coverage to include cancer prevention and treatment into the premium; special provision of funds is also needed to limit the global shortage of human resources in specialist care [30]. This should include prompt remunerations, attractive packages and sponsored cancer care trainings; ensuring availability of equipped diagnostic and cancer care centers across countries, in order to facilitate easy access to care. Access to care will help to reduce loss to follow-up, compliance and adherence issues; Strengthening the institutional cancer registries and integrating them into the NHMIS.

### What is known about this topic

- Cancer is increasing rapidly in African countries and it represents one of the main causes of illness and death among adults;
- Cancer burden is unknown in most of the African countries mainly because of a lack of data or poor reporting of cases. As a result countries rely on estimated data. Health systems face several challenges but they are not well documented and/or assessed;
- Efforts are ongoing to tackle the disease but they are not well designed and tailored to the needs because countries do not have the vital data needed to make informed decisions and policies.

### What this study adds

- The magnitude of the challenges faced by health systems to prevent and control cancer and the capacities that exist in countries;
- What are health systems gaps to prevent and control cancer with regard to the six pillars of health system strengthening namely: leadership and governance, healthcare financing, health workforce, medical products and technology, service delivery and health information;
- What are the key recommendations to be implemented by countries through informed decisions and policies in order to enable health systems to fight against cancer.

## References

[1] World Health Organization, editor (2014). The health of the people: what works: the African regional health report, 2014.

[2] Institute for Health Metrics and Evaluation Global, regional, and national disability-adjusted life years (DALYs) for 359 diseases and injuries and healthy life expectancy (HALE) for 195 countries and territories, 1990-2017: a systematic analysis for the Global Burden of Disease Study 2017.

[3] Freddie Bray, Jacques Ferlay, Isabelle Soerjomataram, Rebecca L Siegel, Lindsey A Torre, Ahmedin Jemal (2018). Nov 2018Global cancer statistics 2018: GLOBOCAN estimates of incidence and mortality worldwide for 36 cancers in 185 countries. CA Cancer J Clin.

[4] WHO Cancer.

[5] The ASCO Post Breast Cancer and Noncommunicable Diseases: Where in the World Do We Start?.

[6] The world bank The Global Burden of Disease: Main Findings for Sub-Saharan Africa.

[7] Sankaranarayanan R (2011). Cancer survival in Africa, Asia, the Caribbean and Central America. Introduction. IARC Sci Publ.

[8] Bollyky TJ, Templin T, Cohen M, Dieleman JL (2017). Lower-Income Countries That Face The Most Rapid Shift In Noncommunicable Disease Burden Are Also The Least Prepared. Health Aff Proj Hope.

[9] World Health Organization, editor (2010). Monitoring the building blocks of health systems: a handbook of indicators and their measurement strategies.

[10] Countryeconomy.com ECOWAS - Economic Community of West African States 2019.

[11] Ghana Health Service Ghana Health Facts and Figures 2017.

[12] Agence Nationale de Statistique et de la Démographie Le Sénégal en bref.

[13] Omoruan AI, Bamidele AP, Phillips OF (2009). Social Health Insurance and Sustainable Healthcare Reform in Nigeria. Stud Ethno-Med.

[14] WHO NCDs | Assessing national capacity for the prevention and control of NCDs.

[15] WHO The Abuja Declaration: Ten Years On.

[16] Kenneth Chima Nwankwo, David A Dawotola, Vinay Sharma (2013). Radiotherapy in Nigeria: Current status and future challenges. West African Journal of Radiology.

[17] Charity Binka, David Teye Doku, Kofi Awusabo-Asare (2017). Experiences of cervical cancer patients in rural Ghana: An exploratory study. PLoS One.

[18] Brunner B, Barnes J, Carmona A, Kpangon A, Riley P, Mohebbi E (2016). Senegal Private Health Sector Assessment: Selected Health Products and Services.

[19] ACCA Key health challenges in Ghana.

[20] Egenti B (2016). Breast Cancer in Nigeria: Diagnosis, Management and Challenges.

[21] Musa J, Nankat J, Achenbach CJ, Shambe IH, Taiwo BO, Mandong B (2016). Cervical cancer survival in a resource-limited setting-North Central Nigeria. Infect Agent Cancer.

[22] Diop M, Kanouté A, Diouf M, Ndiaye A, Lo CMM, Faye D (2017). Behavior of the access to oral health care in Senegal. Edorium J Public Health.

[23] Wiley Online Library Costs, effects and cost-effectiveness of breast cancer control in Ghana.

[24] Dennis Uba Donald (2015). Challenges of Clinical Leadership in Nigeria. Journal of Psychiatry.

[25] Journal of Cancer Science & Therapy A Systemic Review of Incidence of Cancer and Challenges to its Treatment in Nigeria.

[26] Naicker S, Plange-Rhule J, Tutt RC, Eastwood JB (2009). Shortage of healthcare workers in developing countries Africa. Ethn Dis.

[27] Gorman E, Yu S, Alamgir H (2010). When healthcare workers get sick: exploring sickness absenteeism in British Columbia, Canada. Work Read Mass.

[28] Zali MR, Safdari R, Maserat E, Asadzadeh Aghdaei H (2016). Designing clinical and genetic guidelines of colorectal cancer screening as an effective roadmap for risk management. Gastroenterol Hepatol Bed Bench.

[29] Harfouche A, Silva S, Faria J, Araùjo R, Gouveia A, Lacerda M (2017). Breast Cancer: Value-Based Healthcare, Costs and Financing. Acta Médica Port.

[30] Kuehn BM (2007). Global shortage of health workers, brain drain stress developing countries. JAMA.

